# Supplementary material for: Are carbon emissions trading and green financial instruments synergistic? -Comprehensive quantitative research based on content analysis
Source: PLoS One. 2024 Mar 7;19(3):e0298601. doi: 10.1371/journal.pone.0298601 (PMC10919601; doi:10.1371/journal.pone.0298601)
Supplement: S1 Data — (ZIP) [file pone.0298601.s001.zip › Data/地级市Z_F工作报告.zip!/╜¡╦╒/┴1⁄4╘╞╕█╩╨/└┤╫╘╣1⁄2╓┌║┼í╛┬φ┐╦╩2╛▌═°í┐_2007.docx]

各位代表：

现在，我代表市人民政府向大会作工作报告，请予审议，并请各位政协委员提出意见。

2006年工作情况

2006年是实施　十一五规划开局之年，是我市经济社会保持快速发展、呈现明显变化的一年。全市人民在中共连云港市委的领导下，以邓小平理论和三个代表重要思想为指导，全面落实科学发展观，积极顺应国家宏观调控政策，抢抓机遇，奋发进取，坚定不移地向全面小康目标迈进，全面完成了市十一届人大四次会议确定的各项任务。

一、经济健康快速发展，产业实力稳步提升

实施新一轮区域发展战略，加快经济结构调整，经济增速与质量同步提升。完成地区生产总值527亿元，增长15.1%，一二三产分别增长6%、19.2%、15.3%，三次产业结构由20.5∶44.2∶35.3调整为18.2∶45.9∶35.9。完成财政总收入78.3亿元，增长39.2%，其中地方一般预算收入34亿元，增长38.1%。需求有效拉动经济增长，全社会固定资产投资、社会消费品零售总额、外贸出口分别增长34.9%、16.2%和56.6%，经济运行协调性明显增强。

农业综合效益不断提高。扶持引导农业调优调强，现代农业体系加快构建。特色农业区域化布局进一步优化，优质水稻、设施蔬菜、高效渔业、畜禽养殖等特色产业规模不断扩大。粮食总产277.1万吨，高效农业面积达126万亩，高效渔业面积达10万亩，畜禽规模化养殖比重提高6个百分点。成片造林14.5万亩。生态农业加快发展，农产品质量建设加强，20多个无公害、绿色和有机农产品远销海外市场，农产品出口增长42%，20家龙头企业跻身省农产品出口百强。农业产业化经营加快推进，成立市农牧业生产、经营、出口协会，新创办农民专业合作组织43家，东海黄川草莓协会入选全国百家优秀专业农村协会，农产品加工转化率提高6个百分点。农机新机具新技术加快推广，农资农技服务网络逐步健全，农业综合配套体系不断完善。

工业经济实力持续增强。规模以上工业增加值、利税分别增长21.8%和27%。T型产业布局加快展开，临港工业和沿东陇海线制造业快速发展，产业集聚效应初步形成，石化、船舶制造、电子信息等项目取得突破。推进产业结构调整，化工、机械等重点产业加快集聚，新医药、新材料等优势产业竞争力增强。升德升、汉高华威分别入选国家汽车零部件出口基地企业和国家电子信息产业发展基金项目，连众牌玻璃钢管道被评为中国名牌产品。完成工业投资260.5亿元，50项重点项目先后开工，30项重点项目竣工投产，在建亿元以上项目68项。工业新增长点培育成效明显，新增规模以上企业210家，营业收入过10亿元企业8家，100个新增长点项目对工业销售收入增长的贡献率达到76%。建筑市场不断扩大，建筑业增加值增长18.5%。

服务业综合功能快速提升。加快建设区域性国际物流中心，港口集疏运体系、口岸大通关环境不断优化。港口吞吐量7232.2万吨，集装箱运量130.2万标箱，分别增长20.2%和29.5%。民航客运量增长61%。道路运输结构得到调整，水路货物运输加快发展。旅游业持续升温，成为中国优秀旅游目的地之一，云台山风景名胜区列为首批国家重点风景名胜区数字化建设试点，花果山景区入选中国外国人最愿意去的50个地方，新创国家工农业旅游示范点4个。境内外游客接待量、旅游总收入分别增长15%和24.2%。专业市场、连锁经营、电子商务快速发展，城乡市场流通体系进一步完善，社会消费品零售总额211.5亿元，成交额过10亿元市场3家。粮食托市收购70万吨。居民消费价格涨幅1.4%。房地产市场秩序进一步规范，房地产开发面积、商品房销售额分别增长56.2%和25.7%，小区物业管理市场化进程加快。信息资源开发利用、网络普及水平不断提高。优化信贷结构，金融与地方经济良性互动发展，金融机构各项存贷款余额分别比年初增加81.3亿元和71.4亿元，现金收入增长24.4%。保险业务不断拓展，保费收入增长11.7%。

区域发展潜力迅速上升。我市入选首届中国投资环境百佳城市，灌南、东海、灌云跻身全国最具投资潜力中小城市百强，赣榆入围长三角最具投资价值县市。市区发展活力明显增强，市开发区在国家级开发区位次前移5位。县域经济快速发展，四县地区生产总值、财政一般预算收入、全社会固定资产投资分别增长17.3%、51.8%、44.5%，灌南率先实现所有乡镇财政收入过千万元。

二、改革开放持续推进，经济活力不断增强

坚持以市场化为导向，加快推进民营经济发展，新注册个体私营企业3万户，新注册资本90亿元，民营经济增加值增长19%，贡献份额显著提高。市属企业改革完成资产重组2亿元，妥善安置破产企业职工和离退休人员8420人。新型国有资产管理体制不断完善，资本运作能力进一步增强，积极引进BT、BOT等运作模式，培育金港湾投资公司等国有投资主体，金海投资公司设立海外公司。金桥盐业公司划转地方管理。积极推行以联合审批、审批代办、重点联系服务为主要内容的行政审批制度改革。市属生产经营型事业单位改制转企基本完成，首批141家市属社会公益型事业单位人事和分配制度改革全面完成。调整市区财税体制，深化金融机构改革。实施商行合并重组，组建东方、灌云农村合作银行。

招商引资强力推进，注册外资实际到帐3.6亿美元，增长30%，引进内联客方到位资金200亿元，增长32.5%。成功承办苏台、苏北经贸洽谈会和江苏国际农洽会、海外之友联谊会等经贸交流活动。园区建设快速推进，产业承载能力明显增强。各类园区完成基础设施投入40亿元，市开发区临港产业区40平方公里具备项目入驻条件，赣榆开发区跻身长三角最具投资价值开发区十强，连云港化学工业园和灌云、海州、连云经济开发区升格为省级开发区，一批专业特色园区初具雏形。园区产业快速集聚发展，五洲半挂车、风力发电叶片、万海石化等一批大型项目落户临港产业区、柘汪产业区，园区工业增加值占全市工业比重达64%。对外经济贸易快速发展，外贸规模企业增加迅速，机电产品出口增长117.3%。外贸进出口总额达27.3亿美元，增长33.8%。外派劳务营业额1.98亿美元，增长19%。兴办10家境外投资企业，2家企业获对外承包工程经营资格。

三、东部城区率先发展，城乡建设统筹推进

突出海滨城市个性特色，城市规划体系进一步完善。围绕一心三极空间布局，不断拓展发展战略规划成果，海滨新区用海规划获国家批准，中央商务区、连岛等16项详细规划编制完成。启动城市总体规划修编，开展近期重要片区、重点地段、重大设施规划研究，编制花果山片区、海州开发区等20余项控制性规划和城市供热、空港物流等10多项专业规划，完成50个乡镇总体规划和315个村庄建设规划。城市中心城区控制性详规覆盖率达96%，东部城区实现全覆盖。

以港口建设为重点，基础设施建设全面加快。港口15万吨级航道扩建进展顺利，10万吨级和第五代集装箱船舶可乘潮进出港。庙岭三期突堤2个集装箱泊位具备简易靠船条件，墟沟二期5万吨级和墟沟三期7万吨级通用泊位建成试投产，氧化铝、散化肥等专业化泊位开工建设。南疏港通道、中云物流园区加快建设。一体两翼组合港规划布局初步展开，南翼灌河港区、北翼赣榆港区等开展前期论证，11个造船、码头项目落户灌南船舶工业园。实施东陇海铁路电气化改造，连盐铁路纳入国家十一五规划。连盐高速公路和323省道、机场路建成通车，连临高速公路先导试验段奠基，改建国省道200多公里。启动盐灌船闸建设，盐河、玉带河加快疏浚。海堤达标防护工程基本完成，新沂河50年一遇防洪工程加快推进。云台、凤凰变电所等一批输变电工程投入使用。电话扩容、宽带城域网等通信网络设施进一步完善。

以东部城区建设为龙头，国际性海滨城市框架初步拉开。强力推进东部城区率先发展，实施成片拆旧建新，中山西路、大港路、海棠路等道路整治出新，连岛、中央商务区、港湾服务区等节点开发全面启动，海滨新区陆域形成工程启动抛填。突出增强产业承载力，加大基础设施投入力度，临港产业区东方大道等两横七纵路网框架基本形成，板桥工业园道路、水电、通讯设施快速推进，北固山大道、平山北路开工建设。在海一方公园建成开放，东区污水处理厂和配套管网规划建设，一批商业服务项目入驻临港产业区。

以中心城市建设为核心，城乡一体化进程快速推进。投资60亿元实施300项城市建设项目，建成区面积扩大到85平方公里。完成城市西、南出入口拆迁，新建改建人民路、凌州路等30多条城市主次干道，建成新海城区外环路网，东西城区连接更加畅通。市政公用设施不断完善，推进垃圾焚烧热电联产项目，完成西气东输接入工程，发展管道燃气用户7000户。民防应急指挥中心主体封顶，建成人防工程5.6万平方米。深入开展四城同创，大力营造优美舒适的城市环境，被命名为省级园林城市。实施港城大道等64项绿化重点工程，新增城市绿地200公顷。加强环境综合整治，完成美化亮化面积111万平方米，重点地段市容市貌得到改观。加大环卫设施投入，新建改建公厕154座，添置一批道路清洗设施。开通新浦至连云地区公交车，有效治理非法营运行为，城市交通营运秩序明显好转。统筹推进城乡一体化，四县县城彰显特色加快向中等城市发展，一批工业重镇、商贸集镇、文化名镇得到培育，创建一批省新型示范小城镇，试点推动村镇建设，全市城市化率达40.1%。

四、科技教育加快发展，社会事业协调进步

强化科技对经济发展支撑，自主创新能力明显提升。高新技术产业加快发展，165个项目列入国家、省级科技计划，新上省以上火炬计划项目20项，25个产品被认定为国家重点新产品和省高新技术产品，7家企业进入全国50个工业行业创新能力十强。鹰游纺机自主研发碳纤维核心技术及工程化设备获得阶段性成果。恒瑞医药、康缘药业入选国家认定企业技术中心，豪森制药设立国家博士后科研工作站。实现高新技术产业产值76亿元。星火支柱产业快速发展，新上省以上星火计划项目40项，星火龙头企业数居全省首位，东海县被确定为国家首批科技富民示范县。与中科院、清华、北大等院校签订科技合作项目14项。知识产权保护力度加大，专利申请量达719件，增长34%。加大教育投入，办学条件进一步改善。新扩建中小学校舍13.1万平方米，六有工程和125所农村初中四项配套工程基础设施建设全面完成并通过省级验收。初高中段入学率分别达99.8%和90%。新增四星级高级中学1所，创建国家级重点中等职业学校2所、省级职业教育实训基地3个。科教创业园区开工建设。义务教育阶段65.3万名农村学生免缴学杂费8048万元。中小学安全管理工作进一步加强，校园环境明显改善。加强人才队伍建设，引进非师范类本科以上毕业生2093人。

各项社会事业健康发展。对外文化交流活跃，文化建设得到加强。藤花落遗址、海清寺塔列为全国第六批文物保护单位，海州五大宫调入选我国第一批非物质文化遗产名录。市文化艺术中心加快建设，市博物馆建成开放。加快推进广播电视村村通工程，有线电视行政村联网率达85%，新发展有线电视用户9.8万户。市县乡三级传染病疫情实现网络直报全覆盖，市急救中心和中医院门急诊楼投入使用，推进市传染病医院和二院东院区门诊楼建设，建成5家民营医院。竞技体育获省级以上奖牌210枚，其中亚运会金牌1枚。成功承办省十六届运动会皮划艇项目等赛事。市体育中心启动建设。积极开展体育进社区、体育下乡活动，赣榆建成村村有健身工程县。实施母婴安康、关爱农村留守儿童工程。国防建设、民兵预备役和优抚安置工作得到加强，消防施救能力进一步提高。建成国家级防震减灾科普教育基地，修建一批宗教活动场所。农业普查扎实开展，地方志续编有序推进。外事、气象、档案工作积极服务经济社会发展，老龄和关心下一代事业取得新成绩。

人口和计生服务水平不断提高，利益导向机制逐步健全，出生人口性别比升高势头得到遏制，计划生育率达90%，人口自然增长率为6.76‰。连云区被命名为全国计划生育优质服务先进区。强化国土资源管理，耕地保护和土地复垦力度加大，土地市场秩序进一步规范，云台山植被恢复取得阶段性成果，海域动态管理和环境监测进一步加强。坚持环保优先，创优人居生态环境，市区空气质量优良率为88.8%，饮用水源保护得到加强，东海污水处理厂尾水排放工程开工建设，灌云污水处理厂一期工程竣工。创建国家环保模范城市通过国家级技术评估，东海、赣榆通过国家生态示范区验收。

民主法制和精神文明建设扎实推进。定期向人大及其常委会报告工作，向政协通报情况，办理人大代表建议168件、政协委员提案295件。加强与工会、共青团、妇联等人民团体联系沟通，自觉接受社会各方面监督。大力推行政务公开，政府工作规范化、法制化水平进一步提高。认真落实党风廉政建设责任制，建立健全教育、制度、监督并重的惩防体系，发挥监察审计监督职能，深入开展纠风治乱，严肃查处违法违纪行为。平安连云港创建成效明显，被省命名为社会治安安全市和综合治理先进市，七个县区均进入省社会治安安全县区行列。加大信访纠纷排查调处力度，全面推行领导干部下访，信访总量进一步下降。着力提高突发公共事件应对能力，扎实开展安全生产基础年活动，安全生产工作获省政府一等奖。大力整顿和规范市场经济秩序，扎实开展食品药品、农资安全、打击传销等专项整治活动。加强八荣八耻荣辱观教育，推行公民道德规范，积极营造跨越发展氛围和良好社会风尚。举办在海一方暨先进文化进万家系列文化活动，我市雷锋车被命名为全国第一雷锋车，陇海步行街入选第六批全国百城万店无假货示范街，城市文明形象和市民文明素质明显提升。

五、人民生活持续改善，社会保障切实加强

注重提高民生质量，认真解决涉及群众切身利益的突出问题，切实让改革发展成果惠及广大群众。提高职工平均工资、最低工资标准，维护农民工权益，居民收入较快增长，城市居民人均可支配收入11476元，农民人均纯收入4250元，分别增长14.7%和10%。居民消费层次快速提升，文化休闲等消费比重大幅提高，汽车等贵重消费品加速进入家庭，新增私家车9215辆。住房公积金归集7.2亿元，建成经济适用房21.7万平方米，新开发住宅小套型比重70%以上。城乡居民人均住房面积分别达32和38平方米，市区350户特困户享受廉租房政策保障。

认真落实各项惠农政策，新农村建设全面推进。启动双百工程，实施道路、环境、水利、规划和农业综合开发等一批项目。农村新五件实事扎实推进。建成农村公路1600公里，99%行政村通上水泥路或柏油路，农村客运班车通达率95%。农村义务教育、职业技术教育和劳动力转移培训工程协调推进。乡镇卫生院和农村社区卫生服务机构逐步完善。乡镇文化站建设、送戏下乡等工作有效开展。农村河道疏浚、清洁能源推广、卫生改厕等农村环境整治工程顺利推进。实施万村千乡工程，新建改建农家店和农村超市489家，农村商品流通网络初步形成。扶贫开发和老区开发力度不断加大，实施千村万户帮扶工程，投入各类帮扶资金1.7亿元，实现218个经济薄弱村三级帮扶全覆盖，帮助2.4万户农民发展增收项目。

形成新一轮就业政策框架体系，就业再就业工作平台进一步完善，城乡统筹就业扎实推进。开展企业和谐劳动关系创建，劳动合同制度全面推行。再就业优惠政策有效落实，再就业资金使用额8949.3万元，发放小额贷款2438万元，各类职业技能培训24.8万人次。新增就业6.21万人，下岗失业人员和困难人员就业再就业2.77万人，零就业家庭就业得到有效保障。城镇登记失业率3.62%。开展农村劳动力资源普查，实施春雨行动推动农村劳动力转移，新增农村劳动力输出9.1万人。

社会保障功能稳步提升。两个确保得到巩固，城乡居民最低生活保障应保尽保。支付离退休人员养老金9.14亿元、失业保险金6357万元，发放城乡居民最低生活保障金1451万元和4204万元，农村五保户集中供养率50.3%。残疾人等弱势群体基本生活得到保障。扩大医疗、工伤、生育保险覆盖面，1.9万名盐业职工、2.25万名破产企业退休人员纳入医保，推动特困人员、灵活就业人员纳入医保，实施农民工医疗、工伤保险平安计划。新型农村合作医疗参合率达93.6%，四县筹资标准由人均30元提高到50元。赣榆农村合作医疗工作经验在全国推广。新设立7所惠民医院，成立四县慈善总会，发挥市困难职工援助中心和光彩事业促进会作用，加大对特困群体生活医疗救助。

各位代表，过去一年的工作成绩来之不易，这是全市人民在中共连云港市委领导下，解放思想、开拓创新、团结奋斗的结果，是方方面面通力合作、协调配合、大力支持的结果。在此，我代表市人民政府，向全市广大干部群众、驻连部队、武警官兵、公安干警、驻连部省属单位和支持连云港发展的海内外朋友，向各位人大代表、政协委员和各民主党派、工商联、各人民团体、各界友好人士，表示崇高的敬意和衷心的感谢！

回顾过去的一年，我们也清醒地看到，我市经济社会发展中还存在不少薄弱环节，面临的矛盾和困难还很多，主要是经济综合实力还不强，重大产业项目需要进一步突破；对外开放水平还不高，招商引资力度需要进一步加大；新农村建设任务相当繁重，农民持续增收渠道需要进一步拓展；社会服务和公共产品欠帐较多，社会事业发展需要进一步推进；维护社会公平、促进社会和谐任务十分艰巨，社会保障能力需要进一步加强；政府自身建设与跨越发展要求还有差距，经济社会发展环境有待进一步优化。我们必须高度重视并认真解决这些问题，切实把各项工作做得更好。

2007年工作意见

各位代表，2007年是党的十七大召开的重要一年，是我市跨越发展求突破、提升区域整体竞争力的关键一年。今年元旦温家宝总理来连视察时指出，连云港南联长三角，北接渤海湾，隔海东临东北亚，又通过陇海铁路西连中西部地区以至中亚，是连接东西南北的纽带，在我国区域经济协调发展中具有重要战略地位。我们必须进一步增强跨越发展的紧迫感和责任感，体现富民强市的本质要求，推动经济社会发展全面突破。今年政府工作的总体要求是：以邓小平理论和三个代表重要思想为指导，全面落实科学发展观，按照市十次党代会和十届三次全会部署，以全面奔小康，重振连云港为总目标，以好中求快，全面突破为年度工作主题，突出产业集聚和海滨城市建设，突出改革创新和重大项目突破，突出新农村建设和县区发展振兴，突出社会事业发展和民生质量提高，进一步优化发展环境，积极构建和谐社会，创造跨越发展、科学发展、和谐发展的新业绩。

2007年经济和社会发展主要预期目标是：地区生产总值增长15%、力争16%，单位生产总值能耗完成省下达任务，财政总收入突破100亿元，其中一般预算收入增长25%、力争27%，全社会固定资产投资增长25%、力争27%以上，社会消费品零售总额增长15%，外贸出口增长15%、力争20%，实际到帐注册外资增长25%、力争50%以上，居民消费价格涨幅3%以内，城市居民人均可支配收入增长10%，农民人均纯收入增长8%，城镇登记失业率3.7%以内，人口自然增长率7‰以内，城市化水平提高2个百分点。

全市重点抓好十项工程：⑴开工建设25万吨级矿石码头水工主体工程。⑵建成南疏港通道一期工程，开工建设东疏港通道。⑶花果山大道快车道建成通车。⑷建设盐灌船闸闸首闸室主体工程，开工建设204国道新沂河大桥。⑸建成海堤公路达标路段路面工程，加快建设连临高速公路，242省道全线开工。⑹开工建设中云火车客运站、东部城区公交总站。⑺科教创业园建成连云港师专、职业技术学院新校区和716研究所研发基地一期工程。⑻完成体育中心体育馆桩基和地下工程，开工建设主体育场和游泳馆。⑼投资4亿元进一步提升通讯网络容量与覆盖质量。⑽海滨新区完成围海5200亩并抛填3200亩，建成北固山大道、平山北路，开工建设东方国际商务广场等一批项目。

全市切实做好十件实事：⑴城市居民、在校中小学生和破产关停企业职工5万人纳入城镇医疗保险，市区农村居民医疗保险覆盖率85%，全面免除义务教育阶段学杂费，法律援助做到应援尽援。⑵建成覆盖全社会的公共安全社会面监控系统，行政村食品放心店覆盖面达40%，县、乡、村药品配送率达95%。⑶完成城市西、南出入口绿化景观工程，修建便民道路120条，新改建公厕30座，新建50个新农村服务中心。⑷开工建设经济适用房20万平方米、拆迁安置房60万平方米，对市区450户住房困难家庭提供配租保障。⑸开展集中式重点饮用水源环保整治专项行动，开工建设大浦河调尾工程。⑹建成农村公路800公里、改造危桥30座，优化市区公交线路6条。⑺建成全市处置突发事件应急指挥平台，公共事件应急预案实现联网互动。⑻建成郁洲公园。⑼基本建成垃圾焚烧热电项目。⑽建设市一院门诊病房信息综合楼，完成市二院东院门诊急诊综合楼主体工程。

为完成上述目标和任务，着重抓好以下七个方面工作：

一、着力推进产业结构优化

坚持新型工业化方向，充分发挥资源、生态等比较优势，有效放大港口、园区等承载功能，全面提升产业规模和竞争力，不断提高制造业和服务业比重，促进经济结构优化和增长方式转变。

以园区为载体，以产业为依托，以项目为抓手，加快实施沿海开发和沿东陇海线产业带建设。优化空间布局，以石油化工、船舶制造等项目为突破，推动临港大型工业加快向沿海各产业区集聚，引导都市型制造业加快向各类园区集中，着力延伸产业链条，形成产业集聚、企业集群发展效应。实施新一轮高新技术产业翻两番计划，促进新医药、新材料、新能源产业做大做强，加快发展风力发电装备、专用车辆、特种机械等特色装备制造业。加速运用新技术新工艺改造提升化工、食品、纺织、建材等传统优势产业，促进产品结构升级换代。重抓项目建设，扶持骨干工业企业增强综合竞争力。完成工业投资320亿元以上，实施60项重点开工和40项重点竣工项目，确保新增长点项目新增销售突破100亿元，销售收入过10亿元企业10家，工业增加值增长21%。建筑业增加值增长11%。

以生产性服务业为重点，培育壮大现代服务业，提升改造传统服务业。加快完善以港口物流为龙头的现代物流体系，强化港口基础设施、港湾商务区和中云物流园区建设，加快建设口岸公共信息平台和配套设施，进一步提升口岸服务质量，做大配煤、货物集散交易等物流业务。加密近远洋航线航班，增开铁路货运班列，放大公路集装箱运输优惠政策效应，拓展港口物流辐射范围。完成港口吞吐量8500万吨，集装箱运量180万标箱。推进空港口岸开放，开通临时包机。大力发展连锁经营、物流配送等新型商贸业态，拓展农村、社区连锁销售终端网络，农家店发展到1000家。实现社会消费品零售总额240亿元。完成中国优秀旅游城市复核验收，推进新丝绸之路明珠城市申报，实施旅游资源深度开发和保护，温泉度假区和抗日山景区争创AAAA级旅游区。境内外游客接待量、旅游总收入分别增长12%和15%。强化住房保障制度建设，促进房地产市场健康有序发展。改善社区服务设施，构建社区服务良性发展机制。推进国民经济和社会信息化，积极扶持软件业发展，加快地理空间、应急指挥、农村信息化服务等平台建设。培育多元化市场主体，积极承办各类展会活动，着力打造会展经济品牌。改进银企合作模式，引导信贷投向，促进金融服务创新，为地方发展提供有力支撑。

二、扎实推进新农村建设

把发展现代农业作为新农村建设的首要任务，突出高效、生态、外向发展方向，用现代理念引领农业发展，促进产业结构优化。培育农业主导产业，大力推进现代高效农业规模化，新增高效农业种植面积20万亩。加强农产品优势产业和基地建设，着力培育优质水稻、蔬菜、畜禽、林果和高效渔业等五个产业板块。加快科技兴农步伐，健全农业技术推广体系，重视引进推广特色良种和新技术，用现代科学技术改造农业，提高农业水利化、机械化和信息化水平。大力发展生态农业，争取外向型农业新突破，农产品出口增长20%以上。培育壮大龙头企业，延长产业链条，增强市场竞争力和带动能力。

把增加农民收入作为新农村建设的核心目标，广辟增收渠道，不断扩大农民持续增收空间。进一步提升农产品竞争力，增加农民经营性收入。加强资源开发利用，大力发展农产品精深加工，提高农产品附加值，狠抓专业技能、岗位定向培训，推进农村劳务输出百万工程，着力增加农民工资性收入。坚持产业与市场互动，建设一批具有产业支撑、地方特色和较强辐射力的批发市场，有效提高农民进入市场的组织化程度。继续实施扶贫开发、百名教授科教兴百村和民企帮村工程，进一步抓好100个新农村建设示范村和218个经济薄弱村帮扶。

把统筹城乡发展作为新农村建设的动力机制，坚持以工业化致富农民、城市化带动农村、产业化提升农业。引导城市企业、资金、人才三下乡，加快强县富民、兴镇壮村步伐。按照多予少取放活的方针，认真落实各项支农惠农政策，改善农村金融服务，推进农业保险试点，增强农村经济发展活力。加强农村道路联网、水电网改造、通信网络覆盖等基础设施建设，办好涉农教育、文化、卫生等社会事业，实施清洁水源、家园、田园三清工程，创造整洁、卫生、宜人的农村新环境，改善农村生产生活条件。建立健全支农资金稳定增长机制，确保财政支农投入的增量继续高于上年，固定资产投资用于农村的增量继续高于上年，土地出让收入用于农村建设的增量继续高于上年，今年市财政安排三农支出增长39%。

三、强力推进改革开放

坚持以改革创新为动力，大力强化体制机制创新，有效激发内生活力。努力改善创业环境，进一步落实扶持政策，加快发展民营经济，实现增加值增长17%。妥善安置破产企业职工，全面完成市属企业改制任务。有效整合国有资产，提高国有资本运作能力，引导优势企业增资扩股，促进企业在境内外上市。健全国有资产法人治理结构，完善国资监督管理体制。深化投融资体制改革，重点推进商业银行、农村信用社、邮政储蓄等金融机构改革。优化金融生态环境，创新信用体系，努力增强地区融资能力，壮大金融担保机构实力，改善中小企业融资条件。完善市、区财政体制，推进政府收支分类改革。归并整合、精简规范行政管理型事业单位，推进社会公益型事业单位综合配套改革，基本完成县区生产经营型事业单位转制改企。开展乡镇综合配套改革试点。

创新思路强力推进招商引资，引进外部优质资源推动跨越发展。以规划引领招商，按照生产力布局和产业规划优选招商重点和方向，引导产业对接。以资源引领招商，整合盐田滩涂等特色优势资源瞄准重大投资主体，承接项目落户。以环境引领招商，加快完善基础设施，打造一流投资发展软环境，吸引企业入驻。突出重大项目招商，精心策划包装龙头型、基地型大项目，健全跟踪服务和协调推进机制，形成重大项目在谈在建、储备推进良性循环。突出产业链招商，围绕石化、汽车、造船、硅资源和现代服务业等产业，促进上下游产业延伸和集聚，加快构筑产业集群。突出企业招商，鼓励引导企业发挥自身优势靠大联强，借助外力迅速扩大增量，形成招商引资的连锁效应和群体效应。继续做好国际农洽会等招商经贸活动。力争实际到帐外资5.5亿美元，确保内联到位资金250亿元。

以临港开发为重点，加快推进园区建设。按照规划科学、布局合理、功能配套、特色鲜明、环保生态的要求，加大综合设施投入，提高园区产业承载力。市开发区临港产业区76平方公里、板桥工业园10平方公里具备项目进区条件，加快柘汪、海头、灌河口等两翼临港产业区开发，启动日韩工业园建设。着力提升园区科技企业孵化器功能，提高园区投资强度和产出效益，建立园区投入产出评价考核体系。各类园区基础设施投入46亿元，工业增加值占全市工业比重提高5个百分点。

优化对外贸易结构，壮大外贸出口主体，大力扶持高技术含量和自主品牌产品出口，增加机电产品出口比例，扩大本地产品出口。引进高附加值加工贸易项目，促进加工贸易转型升级，建立一批大宗农副产品出口基地，加快发展国际服务贸易。利用国家双边贸易协议和自由贸易区政策，促进企业拓展国际市场。力争完成外贸出口17.5亿美元。推动境外劳务健康发展，支持优势企业开展境外投资，推进金海公司等境外项目建设。

四、加快建设国际性海滨城市

加大规划编制整合力度，完成新一轮城市总体规划纲要和云台山风景名胜区总体规划修编，深化分区规划和修建性详规，编制物流园区、综合交通等15项控制性详规，抓好经典示范区、标志性建筑和城市节点设计，完善重大基础设施专业规划，加强公共设施、医疗、教育、文化等专项规划，加快村镇建设规划编制，基本实现城乡规划在层次、功能、管理上的全覆盖，统筹推进海滨城市建设。创新规划管理模式，放开规划设计市场，健全公众参与机制，提高规划编制水平。

围绕增强城市集聚辐射功能，突出抓好交通、水利、电力、通讯等30项重大项目。推进一体两翼组合港建设，加快连云港区15万吨级深水航道扩建，续建墟沟、庙岭、旗台港区等大型深水泊位，建设客运站主体工程。加快燕尾港疏港公路建设，开工建设灌河散货码头和船业码头，开展灌河口拦门沙治理研究，推进赣榆港区新奥工业码头项目研究。加快港口集疏运通道建设，改建310等国省道。完成东陇海铁路电气化改造。启动疏港航道工程建设，完成市区盐河疏浚，加快完善内河水运体系。推进新沂河50年一遇防洪工程，实施一批河道整治和水库除险加固工程。建设通信监控综合楼和北崮、包庄变电所等一批输变电工程。新增人防工程5万平方米，城市人均面积达到0.5平方米，民防应急指挥中心投入使用。

加快实施城市东进战略，全面推进港湾商务区、滨海居住区等六个组团和临港产业区、板桥工业园等产业集聚区建设。港湾商务区加快建设地标建筑，西墅海滨新区规划建设高端商务区及住宅区。临港产业区实施7项景观工程和9项沿路绿化工程，基本完成基础设施建设。启动板桥工业园二期15平方公里抛填工程。建设海滨城市主轴花果山大道，加快平山广场、连徐高速公路起点处等节点建设。新海城区推进东进北连，加快凤凰、东河、孔望山等片区开发。城市建成区面积突破90平方公里。推进旧城改造，完成拆旧建新120万平方米，继续实施主干道沿街平改坡工程。优化城市路网结构，加快建设新建东路、凌州东路等20条城市主次干道。围绕创建国家园林城市，加快推进城市绿化，新增城市绿地面积220万平方米。加强城市精细化人性化管理，推进城管进社区、下乡镇。强化城市交通营运秩序整治和违法建设查处。完成主次干道、窗口地段公厕布点。综合整治市容环境，加大城中村、城郊结合部和农村环境整治力度，推进垃圾无害化处理，促进城乡社会环境整体改善。

统筹推进县城、重点中心镇和示范村建设，加速构建现代城镇体系。加大四县县城市政设施、住宅小区、生态环境建设力度，提升产业支撑力和辐射带动功能。赣榆积极融入中心城市组团，东海、灌云、灌南彰显特色扩张功能，加快向中等城市迈进。推进重点中心镇、特色小城镇和农民集中居住点建设，引导农村人口向城镇集中、工业企业向园区集中、居住生活向小区集中，加快城乡一体化进程。

五、大力实施科教兴市战略

着力推进自主创新和科技创业，加快建设创新型城市。以企业为主体，以园区和基地为载体，以科研院所为依托，努力提升高新技术重点领域自主创新能力，推进重大科技成果转化，实现高新技术产业集聚。组织实施火炬计划项目20项，开发省高新技术产品和国家重点新产品30个，高新技术产业产值90亿元。强化4个国家级特色产业基地建设，加快培育新能源、现代装备制造、电子信息产业群，5个项目列入国家和省重大科技计划。推动星火支柱产业技术升级，组织实施星火计划40项，实施农业科技攻关和科技成果示范推广计划30项。加强产学研联合，继续扩大与中科院、清华大学、国防科工委的交流合作，促进科技成果产业化。强化企业科技创新主体地位，加强科技创业服务中心建设，优化科技创业服务。推进知识产权工作体系建设，加强知识产权保护，专利申请量800件。

坚持教育优先，促进各级各类教育协调发展。继续加大教育投入，今年市财政安排教育支出1.27亿元，增长26%。进一步改善办学条件，改扩建校舍20万平方米。提高义务教育水平，启动建设农村小学四项配套工程，完成125所农村初中四项配套工程。加强高中内涵建设，扩张优质高中教育资源。大力发展职业教育，整合中等职业教育资源，加强骨干学校和实训基地建设，创建省示范性职业院校和国家级重点中等职业学校各1所。加快发展高等教育，支持淮海工学院扩大办学规模提高办学水平。加强成人教育和社区教育。实施关爱农村留守儿童工程，健全教育资助体系，确保贫困家庭学生不因贫失学。加大人才培养力度，提高人才队伍素质，围绕港口建设、产业发展、国际经贸等重点领域，引进本科以上紧缺人才2400名。

六、积极推进和谐社会建设

坚持富民优先原则，奠定和谐社会基础。在经济发展中更加关注民生，不断改善民生条件，提高民生质量。扎实开展全面小康进程监测工作，千方百计增加城乡居民收入，注重解决群众就业、社会保障等实际问题，切实维护社会公平正义。以产业经济快速发展有效提升居民收入层次，以积极促进创业多渠道增加居民收入来源，以合理调整分配关系逐步缩小居民收入差距，以完善社会保障体系、发展社会福利事业确保困难群众基本生活。继续落实积极的就业政策，多渠道开发就业岗位，加强职业技能培训，提高劳动者从业素质，促进就业再就业，重点帮助困难家庭人员就业。今年市财政安排社会保障支出7220万元，增长38%。推进城市居民医疗保险全覆盖，开展新型农村养老保险制度改革试点，做好失地农民、困难家庭、残疾人等弱势群体基本生活保障工作。

推进社会事业发展，营造和谐社会环境。今年市财政安排2.95亿元支持社会事业发展，增加5090万元。加强社会文化建设，构建公共文化服务体系。开展国家、省文化先进县区创建工作，启动全市文物普查工作，建立市首批非物质文化遗产保护名录和保护中心，推进海清寺重建，市少儿活动中心投入使用，加快市妇女活动中心建设。推进广播电视村村通工程，新发展有线电视用户8.5万户，农村有线电视行政村联网率92%。实施锦屏山电视发射台改造，实现广播电视节目网上直播。突出卫生事业公益性、公平性和服务性，城市社区医疗服务人口覆盖率80%以上，农村社区卫生服务机构健全率达70%，传染病总发病率低于全省平均水平。继续推进万村体育健身工程，积极备战第六届全国城运会。抓好国防教育和国防动员，扎实开展军民共建活动，争创全国双拥模范城五连冠。抓好外事侨务和对台事务，做好气象、防震减灾、新闻出版工作，重视发展老龄和关心下一代事业。

统筹人与自然协调发展，强化和谐社会保障。稳定低生育水平，提高人口素质，开展省人口协调发展先进县区创建活动，计划生育率90%以上。加快建设环境友好型和资源节约型社会。以建设生态市为目标，建立防治污染长效机制，加强环境保护和污染整治，加大环境修复力度。加强国土资源保护和合理利用，保护海洋生态环境，促进自然资源系统和社会经济系统的良性循环。大力发展循环经济，有效节约资源能源。

加强民主法制和精神文明建设，创建和谐社会秩序。定期向人大及其常委会报告工作，坚持重大问题与政协协商，认真办理人大代表建议和政协委员提案，密切与各人民团体的联系，完善基层民主制度。加强法制宣传教育，规范政府行政措施，全面推进依法行政，加快建设法治连云港。推进科技强警示范城市建设，加强社会治安综合治理，扎实创建平安连云港。进一步加强安全生产基础工作，强化企业主体责任，预防重特大事故发生。全面落实信访责任制和领导干部下访制度，畅通信访渠道，从源头上预防和化解社会矛盾。继续实施公民道德建设工程，深入开展社会主义荣辱观教育实践，完善学校、家庭、社会三位一体的未成年人思想道德教育体系，广泛开展创文明社区，建和谐社会活动，营造和谐奋进、争先创优风尚，提升城市文明内涵和社会文明程度。

七、切实提升政府行政能力

面临跨越发展的新形势新任务，必须坚持立党为公、执政为民的宗旨，推进政府管理创新，加强行政能力建设，努力做到权为民所用、情为民所系、利为民所谋。

提升行政执行能力。建立健全层级清晰的责任体系，强化行政责任。提高行政效能，做到政府各项决策和任务执行到位，确保政令畅通和高效运转。抓好执法队伍建设，进一步加强在环境保护、城乡建设、安全生产、劳动保护等领域的执法监管。完善决策事项听证论证、公众参与、合法性审查和责任追究制度，保证决策合法合规、顺应民意、切实可行。完善行政投诉机制，强化行政服务中心一站式服务功能。加强激励约束和绩效评估，进一步规范公务员管理。

提升公共服务能力。坚持执政为民，切实履行政府服务职责，着力解决事关群众切身利益问题。重点抓好公用设施、医疗卫生、社会保障等公共服务体系建设，提供更多公共产品，提高电子政务应用水平，为企业和群众提供优质服务。优化配置公共资源，注重向农村、基层、弱势群体倾斜，逐步形成惠及全体市民的公共服务体系。建立健全自然灾害、事故灾难和社会安全事件等应急管理体系，提高处置突发公共事件能力。落实市场监管职能，确保食品药品、医疗、农资等质量安全，建立规范的市场竞争秩序。

提升从严治政能力。把维护群众利益作为从严治政的工作重点，着力解决损害群众利益的突出问题。继续抓好领导干部的教育、监督和廉洁从政，推进惩治和预防腐败体系建设，深入开展党风廉政建设和反腐败斗争。坚决纠正部门和行业不正之风，认真查处投资者和群众反映强烈的突出问题。扎实开展治理商业贿赂工作，加大对工程建设、土地出让、产权交易等领域商业贿赂的查处力度。加强监察审计监督，严格执行领导干部经济责任审计制度。切实改进工作作风，树立为民、务实、清廉的政府形象，提高政府公信力。

各位代表，新的形势催人奋进，新的征程任重道远。让我们紧密团结在以胡锦涛同志为总书记的党中央周围，全面落实科学发展观，在中共连云港市委的领导下，开拓创新，真抓实干，以跨越发展的新成绩迎接党的十七大胜利召开！
